# Survey on medicinal plants and spices used in Beni-Sueif, Upper Egypt

**DOI:** 10.1186/1746-4269-7-18

**Published:** 2011-06-27

**Authors:** Sameh F AbouZid, Abdelhalim A Mohamed

**Affiliations:** 1 Department of Pharmacognosy, Faculty of Pharmacy, Beni-Sueif University, P.O. Box 62111, Beni-Sueif, Egypt; 2 Department of Flora & Phyto-Taxonomy Researches, Horticultural Research Institute, Agricultural Research Centre, P.O. Box 12311, Giza, Egypt

## Abstract

**Background:**

This study was conducted to identify medicinal plants and spices used for medicine by the community of Beni-Sueif, Upper Egypt.

**Methods:**

Ethnobotanical data from local people was collected using direct interviews and a semi-structured questionnaire.

**Results:**

Forty-eight plant species belonging to twenty-seven families and forty-seven genera were encountered during the study. Their botanical and vernacular names, plant parts used and medicinal uses are given. Results of the study were analyzed using two quantitative tools. The factor informant consensus indicated the agreement in the use of plants and the fidelity level indicated the ratio between the number of informants who independently suggested the use of a species for the same major purpose and the total number of informants who mentioned the plant for any use. The results of the factor informant consensus showed that the cardiovascular category has the greatest agreement, followed by the immunological, gastrointestinal and respiratory categories. The most important species according to their fidelity are: *Hibiscus sabdariffa *L. for the cardiovascular category; *Trigonella foenum-graecum *L. for the immunological category; *Mentha piperita *L. for the gastrointestinal category and *Pimpinella anisum *L. for the respiratory category.

**Conclusions:**

Medicinal plants are still used for treatment in Beni-Sueif community despite the availability of prescribed medications. Documentation of this ethnomedicinal knowledge is important. Evaluation of pharmacological activity for the promising medicinal plants is suggested.

## Background

The use of medicinal plants has occurred in Egypt since Pharaonic times [1,2]. This constituted an integral part of the practiced medicine at that time. Nowadays, Egyptians still depend of medicinal plants for treatment. In a recent study conducted by the Information and Decision Support Centre in Egypt [3], it was found that 23% of the Egyptian use medicinal plants as a remedy; 52% of them are living in urban areas and 48% are living in countryside. It is important to document these uses and perform studies about their pharmacological activities to assure their efficacy and safety.

Beni-Sueif is situated in the north of Upper Egypt and occupies a land area of approximately 10954 km^2^. The governorate has a total inhabitancy of 1369.41 km^2^. It boasts a population of over 2315512. The language of inhabitants is Arabic. Biomedical facilities and prescription medication are available in the towns of the governorate in addition to the herbalist shops. The latter represents the main healthcare facility in the countryside. Beni-Sueif has different phytogeographical regions; the desert regions of the western and eastern sides of the Nile and the Nile-valley characterized by its fertile soil [4]. Beni-Sueif governorate is producing 25% of medicinal and aromatic plants produced in Egypt. This is mainly because of the presence of suitable climate, fertile soil and availability of irrigation water from the Nile. *Pelargonium roseum *L., *Ocimum basilicum *L., *Artemisia herba-alba *Asso., *Mentha piperita *L., *Coriandrum sativum *L., *Anethum graveolens *L., *Origanum majorana *L. and *Jasminum officinale *L. are the most important medicinal and aromatic plants produced in the governorate. Table 1 shows the production figures for these plants.

**Table 1:** Medicinal and aromatic plant production in Beni-Sueif, Egypt

Plant	Area (in Fadden)	Production (ton/year)
***Pelargonium roseum *L**.	3071	56137
***Ocimum basilicum *L**.	1724	2586
***Artemisia herba-alba *Asso**.	1334	1100
***Anethum graveolens *L**.	262	2162
***Mentha piperita *L**.	212	262
***Coriandrum sativum *L**.	127	117
***Jasminum officinale *L**.	53	159
***Origanum majorana *L**.	31	26

Data source: Investors Service Center, Beni-Sueif governorate http://www.benisuef.gov.eg, 2004.

The present study aims to review traditional ethnomedicinal knowledge of the local people in Beni-Sueif governorate, focusing specifically on the medicinal uses of plants. These types of studies are urgent considering the loss of traditional knowledge accompanying alteration of the physical and biological environment.

## Methods

### Data collection

**Figure 1:**
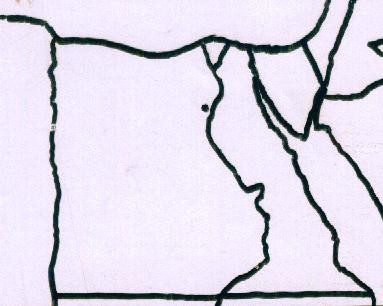
**Map of Egypt**. A map of Egypt showing the location of Beni-Sueif governorate in north part of Upper Egypt.

Direct interviews with the people living in Beni-Sueif governorate (Figure 1) were conducted during the period March-August 2009, using a semi-structured questionnaire [5]. The participants in the interview were chosen from those who declared to know and/or use medicinal plants by a random sampling technique. Recruiters visited participants in their homes and asked them the questions in the questionnaire. Examples of some commonly used medicinal plants were given to make the idea of the study easier for the participants. The participants were asked whether they prefer treatment with medicinal plants to conventional drug therapy. The interview was done in Arabic, the language spoken by the people living in the governorate. The information was collected from 57 persons (44 men and 13 women) whose age ranged from 30-70 years. This includes questions about the plants they use against diseases, parts of the plants used, methods of preparation and sources they obtain these plants. People interviewed are representing urban and rural life styles. The plant materials were obtained from their sources, botanically identified [4,6] and voucher specimens were stored in CAIM herbarium, Flora & Phyto-taxonomy Researches Department, Horticultural Research Institute, Agricultural Research Centre, Ministry of Agriculture, Egypt.

### Quantitative ethnobotany

The factor informant consensus (F_IC_) [7] was used for the analysis of the general use of plants. The illnesses were classified into broad disease categories (several diseases based on the organ system in one category) using the following categories: (1) urological (inflammation, calculi, infection), (2) gastrointestinal (mal digestion, ulcer, flatulence, constipation, colic), (3) respiratory (cough, congestion), (4) neurological (anxiety), (5) cardiological (hypertension), (6) dermatological (infection, wounds), (7) immunological (susceptibility to infections), (8) cultural (spices, condiments, heath drink), (9) diabetes, (10) women's health (menstrual pain, hair growth). The F_IC _was calculated as the number of use citations in each category (nur) minus the number of species used (nt), divided by the number of use citations in each category minus one [7]:

The fidelity level (FI), which is the ratio between the number of informants who independently suggested the use of a species for the same major purpose and the total number of informants who mentioned the plant for any use, was calculated for the most frequently reported diseases or ailments for the categories with the highest F_IC_

Where Np is the number of informants that claimed a use of a plant species to treat a particular disease, and N is the number of informants that used the plant as a medicine to treat any given disease [8].

Number of uses mentioned (Um) refers to the mentions for one plant given by all of the informants for a specific disease.

These tools helped us to determine which illness categories had higher representation (using the F_IC_) and the plants with major fidelity (using the FI).

## Results

### General analysis of the data

The results of the study are presented in Table 2, in which the plants are arranged in alphabetical synopsis. For each species, the following information is provided: botanical name, plant family, local name, part used, method of preparation or administration and ailments treated. Forty-eight plant species distributed over twenty-seven families and forty-seven genera were discovered to be used by local people of Beni-Sueif. Apiaceae, Fabaceae and Lamiaceae were the most dominant families.

**Table 2:** Medicinal plant species, plant parts used and ailments treated by local people of Beni-Sueif, Egypt

Plant name Voucher specimen code Used part	Preparation	Use/ailment treated (No. of uses mentioned, FI)
***Allium cepa *L. (Alliaceae), Bassal, cultivated, CAIM101, B**	Decoction, green parts	Heart diseases (1, 50), antiseptic for mouth infections (1, 50)
***Allium sativum *L. (Alliaceae), Thawm, cultivated, CAIM102, B**	Chewing fresh bulbs after removing external scales	Memory loss (1, 20), hypertension (2, 40), microbial infections (2, 40)
***Ambrosia maritima *L., (Asteraceae), Damsis, wild, CAIM110, L**	Decoction	Renal colic (4, 100)
***Ammi visnaga *(L.) Lam. (Apiaceae), Khillah, cultivated, CAIM103, S**	Decoction	Diuretic (1, 100)
***Amomum cardamom *L. (Zingiberaceae), Habbahan, imported, CAIM144, Fr**	Entire fruits	For general well-being (3, 100)
***Artemisia herba-alba *Asso. (Asteraceae), Shihh, wild, CAIM111, L, F**	Decoction	Colic in GIT (2, 100)
***Boswellia carterii *Birdw. (Burseraceae), Lubn dhakar, imported, CAIM113, G**	Chewing	Expectorant (4, 100)
***Camellia sinensis *L. Kuntze, (Theaceae), Shay akhdar, imported, CAIM142, L**	Infusion	For diet purpose (3, 100)
***Capsicum annum *L. (Solanaceae), Fulful ahhmar, imported, CAIM140, Fr**	Powder	Condiment (1, 100)
***Carum carvi *L. (Apiaceae), Karawya, cultivated, CAIM104, Fr**	Decoction	Colic in GIT (3, 75), hypertension (1, 25)
***Caryophyllus aromaticus *L. (Myrtaceae), Qurunfil, cultivated, CAIM130, Fb**	Buds are placed on pain site	Toothache (2, 100)
***Cassia acutifolia *Delile. (Fabaceae), Sana makki, imported, CAIM118, L**	Decoction	Constipation (6, 100)
***Ceratonia siliqua *L. (Fabaceae), Carob, cultivated, CAIM119, Fr, I**	Infusion	Diarrhea (3, 100)
***Cinnamomum verum *J. Presl (Lauraceae), Qirfah, imported, CAIM124, Bk**	Decoction	General well-being (1, 33.3), menorrheal pain (1, 33.3), colic in GIT (1, 33.3)
***Citrus limon *(L.) Burm. F. (Rutaceae), Laymun, cultivated, CAIM137, Fr**	Juice	Common cold (6, 66.7), anxiety (3, 33.3)
***Cladium mariscus *(L.) Pohl (Cyperaceae), Hhalfa, wild, CAIM115, L**	Decoction	Colic in GIT (1, 100)
***Coffea arabica *L. (Rubiaceae), Bunn, imported, CAIM138, Be**	Powder	For healing of wounds (1, 100)
***Commiphora myrrha *Engl. (Burseraceae), Murr, imported, CAIM114, G**	Chewing	Cough (1, 100)
***Coriandrum sativum *L. (Apiaceae), Kuzbarah, cultivated, CAIM105, Fr, L**	Powder, green herb	Condiment (1, 50), dizziness (1, 50)
***Cuminum cyminum *L. (Apiaceae), Kammun, cultivated, CAIM106, Fr**	Powder, decoction	Condiment (1, 50), colon colic (1, 50)
***Cyperus longus *L. (Cyperaceae), Su'd, wild CAIM116, L**	Powder	Decreasing hair growth (1, 100)
***Foeniculum vulgare *Mill. (Apiaceae), Shamar, cultivated, CAIM107, Fr**	Decoction	Colic in GIT (2, 100)
***Glossostemon bruguieri *Desf., (Sterculiaceae), Moghat, imported, CAIM141, Rt**	Powdered roots are cooked in margarine, and then water is added with continuous stirring. Taken with nuts and coconut.	For general well-being (4, 100)
***Glyccyrrhiza glabra *L. (Fabaceae), Erq sus, imported, CAIM120, Rt**	Moistened powder in a gauze, then diluted with water.	Peptic ulcer (3, 42.9), anti-inflammatory (4, 57.1)
***Hordeum vulgare *L. (Gramineae), Sha'ir, cultivated, CAIM123, Be**	Decoction	Renal colic (2, 66.7), colon problems (1, 33.3)
***Hibiscus sabdariffa *L. (Malvaceae), Karkareeh, cultivated, CAIM131, F**	Decoction or infusion	Hypertension (36, 97.3), antimicrobial (1, 2.7)
***Hyphaene thebaica *(L.) C. Martius, (Palmae), Doum, wild, CAIM134, Fr**	Decoction, entire fruits, powder	Hypertension (2, 50), dyspepsia (1,25), cardiotonic (1, 25)
***Lupinus albus *L. (Fabaceae), Turmes murr, cultivated, CAIM121, S**	Powder	Antiseptic for skin (2, 100)
***Matricaria recutita *(L.) Rauschert, (Asteraceae), Babunag, cultivated, CAIM112, F**	Decoction, powder	Insomnia (1, 25), colon colic (1, 25), cough (1, 25), antiseptic for skin (1, 25)
***Mentha piperita *L. (Lamiaceae), Na'na, cultivated, CAIM125, L**	Decoction	Colic in GIT (28, 87.5), anxiety (4, 12.5)
***Myristica fragrans *Houtt. (Myristicaceae), Gawz et tib, imported, CAIM132, S**	Powder	Condiment (1, 100)
***Nigella sativa *L. (Ranunculaceae), Habbet el barakah, cultivated, CAIM139, S**	Powder, entire seeds	Immuno-stimulant (2, 50), condiment (2, 50)
***Orchis hircine *Crantz. (Orchidaceae), Sahhlab, imported, CAIM133, Rt**	Powdered rots are boiled in milk with continuous stirring	Peptic ulcer (1, 100)
***Origanum majorana *L. (Lamiaceae), Bardaqush, cultivated, CAIM126, L**	Decoction	Stomach troubles and coli (1, 50), hypertension (1, 50)
***Petroselinum sativum *Hoffm. (Apiaceae), Baqdunis, cultivated, CAIM143, L**	Green leaves in salad	Urinary tract infection (2, 100)
***Pimpinella anisum *L. (Apiaceae), Anisun, cultivated, CAIM108, Fr**	Decoction, infusion	Renal colic (2, 5.9), colic in GIT (11, 32.4), upper respiratory tract problems (19, 55.9), anxiety (2, 5.9)
***Piper nigrum *L. (Piperaceae), Fulful aswad, cultivated, CAIM146, Fr**	Powder	Condiment (1, 100)
***Plantago afra *L. (Plantaginaceae), Bazr qatuna, cultivated, CAIM135, S**	Powder	Antiseptic for skin (1, 100)
***Portulaca oleracea *L. (Portulacaceae), Riglah, wild, CAIM136, L, S**	Painful joints are exposed to steam evaporating from boiling leaves with water, powdered seeds	Rheumatic pain (2, 50), stomachic appetizer (2, 50)
***Psidium guajava *L. (Myrtaceae), Guwafah, cultivated, CAIM147, L**	Decoction	Cough and cold (3, 100)
***Rosmarinus officinalis *L. (Lamiaceae), Hassa el-ban, cultivated, CAIM127, L**	Infusion, volatile oil	Carminative (1, 33.3), diuretic (1, 33.3), antiseptic (1, 33.3)
***Salvia officinalis *L. (Lamiaceae), Maryamiyah, cultivated, CAIM128, L**	Infusion	Colic in GIT (3, 60), antiseptic (1, 20), common cold (1, 20)
***Solenostemma argel *Hayne (Asclepiadaceae), Hhargal, wild, CAIM109, L**	Infusion	Antispasmodic for renal colic (2, 100)
***Tamarindus indica *L. (Caesalpiniaceae), Tamer Hindi, imported, CAIM117, Fr**	Decoction	Laxative (3, 100)
***Thymus vulgaris *L. (Lamiaceae), Za'tar, cultivated, CAIM129, L**	Powder	Condiment (4, 100)
***Tilia sylvestris *Desf. (Tiliaceae), Zayzafun, imported, CAIM148, L, F**	Decoction	Cough due to common cold (2, 100)
***Trigonella foenum-graecum *L. (Fabaceae), Hhelbah, cultivated, CAIM122, S**	Decoction	Diuretic (1, 4), colic in GIT (14, 56), nutritive (9, 36), diabetes (1, 4)
***Zingiber officinale *Roscoe (Zingiberaceae), Zangabil, imported, CAIM145, R**	Decoction	Voice problems in common cold (4, 100)

B = Bulb, Be = Beans, Bk = Bark, F = Flowers, Fb = Flower buds, Fr = Fruits, G = Gum, GIT = Gastrointestinal tract, L = Leaves, R, = Rhizome, Rt = Roots, S = Seeds.

The informants in this study use plants for three main reasons: (1) medicinal plants are safer than prescribed medications (81%), (2) medicinal plants are cheaper than prescribed medications (11%) and (3) medicinal plants are easier to get from herbalist shops widely available in rural areas (8%). Only 3% of the informants were against the use of medicinal plants as remedy because of their unknown composition, prescribed medications are more effective and faster in their actions or medicinal plants are not prescribed by the physicians. The plants with the highest number of uses mentioned for any disease were *Hibiscus sabdariffa *L. (37), *Pimpinella anisum *L. (34), *Mentha piperita *L. (32) and *Trigonella foenum-graecum *L. (25). The complete data are presented in Table 2.

### Factor informant consensus and fidelity level

The results of the F_IC _showed that the cardiovascular category had the greatest agreement with an F_IC _of 0.88, followed by immunological disorders (0.80), gastrointestinal disorders (0.78), respiratory disorders (0.78) and neurological disorders (0.50). Within the cardiovascular category, the main reported ailment was hypertension (36 reports). Within the gastrointestinal category, there were 28 reports of colic. Within the respiratory category, there were 19 reports of cough.

We analyzed the categories with the major agreements to indicate the most important plants in each category. For the cardiovascular category, we found that the most important species, according to fidelity, was *Hibiscus sabdariffa *(FI = 97.3). *Trigonella foenum-graecum *(FI = 36) was the most important species for the immunological category. The most important plants in the gastrointestinal category were *Mentha piperita *(FI = 87.5), *Trigonella foenum-graecum *(FI = 56) and *Pimpinella anisum *(FI = 32.4). *Pimpinella anisum *(FI = 55.9) was the most important plant in the respiratory category.

### Correlation between number of uses mentioned and fidelity level

The categories of the plants with higher number of uses mentioned (for one purpose) were correlated with their fidelity level (Table 2). The plants with higher number of uses mentioned for all categories were *Hibiscus sabdariffa *with 36 mentions for hypertension (FI = 97.3), *Mentha piperita *with 28 mentions for colic in gastrointestinal tract (FI = 87.5), *Trigonella foenum-graecum *with 14 mentions for colic in gastrointestinal tract (FI = 56) and *Pimpinella anisum *with 19 mentions for upper respiratory tract problems (FI = 55.9). Some plants showed a high fidelity level (FI = 100) for one ailment such as *Ambrosia maritima *L. for renal colic (4), *Boswellia carterii *Birdw. as expectorant (4), *Cassia acutifolia *Delile. for constipation (6) and *Tilia sylvestris *Desf. for cough (4). The following plants were mentioned once that is considered of low fidelity: *Ammi visnaga *(L.) Lam. as diuretic, *Capsicum annum *L. as a condiment, *Cladium mariscus *(L.) Pohl for colic in gastrointestinal tract, *Coffea arabica *L. for healing of wounds, *Commiphora myrrha *Engl. for cough, *Cyperus longus *L. for decreasing hair growth, *Myristica fragrans *Houtt as a condiment, *Orchis hircine *Crantz. for peptic ulcer, *Piper nigrum *L. as a condiment and *Plantago afra *L. as antiseptic for skin.

## Discussion

Documentation of ethnobotanical knowledge is important to study/understand human-plant relationships [9], implement general policies about the use of natural resources [10] and assess potential livelihood and monetary benefits [11,12].

In this work, we used two quantitative tools to study the main medicinal plants and spices used by the local people in Beni-Sueif governorate. With the F_IC_, the main categories of health conditions for which plants are used, are detected, and with the FI, we selected the most important species from these categories. The cardiovascular, immunological, gastrointestinal and respiratory categories used the most plants.

The FI and the number of uses mentioned support the F_IC_. This is indicated by the observation that the cardiovascular category has the highest F_IC_. This means that the cardiovascular illnesses have the greatest agreement among the informants for being treated by medicinal plants. *Hibiscus sabdariffa*, the most used plant in the cardiovascular category, has the highest FI and number of uses mentioned among all of the plants. Therefore, the F_IC _is a good analytical tool to select categories of illness when analyzing the data as they are presented here.

If we consider FI and the use mentions to analyze the most important plants, we get the following plants: *Hibiscus sabdariffa *for hypertension, *Mentha piperita *for colic in gastrointestinal tract, *Trigonella foenum-graecum *for colic in gastrointestinal tract and *Pimpinella anisum *for upper respiratory tract problems. These plants are cultivated plants and widely used in the Egyptian folk medicine [13]. There are a lot of studies about the pharmacological activities of these plants; *Hibiscus sabdariffa *[14-18], *Mentha piperita *[19], *Trigonella foenum-graecum *[20,21] and *Pimpinella anisum *[22,23]. We have decided to present here the most significant and important studies related to the uses mentioned in our study. The uncommon ethnopharmacological uses of *Glossostemon bruguieri *Desf., *Lupinus albus, Orchis hircine *and *Portulaca oleracea *L. deserves phytochemical and pharmacological studies to examine the reported activities. Some of the uses mentioned are in agreement with those previously reported in the Mediterranean region [24]. For example, *Portulaca oleracea *was reported to be used in muscular-skeletal diseases and for nutritional purposes in Albania, *Ammi visnaga *for kidney and respiratory diseases in Algeria and Egypt, *Coriandrum sativum *as digestive in Algeria, *Foeniculum vulgare *as digestive in Algeria and Cyperus, *Solenostemma argel *for kidney diseases in Egypt, *Mentha piperita *for mental-nervous disorders in Algeria, *Hibiscus sabdariffa *for cardiovascular disorders in Egypt.

## Conclusions

The current study targeted the medicinal plants used by local people of Beni-Sueif, Upper Egypt. Beni-Sueif is a biodiversity area rich of wide-variety of plant species. The calculated F_IC _and FI values were used as a tool to understand which illnesses are preferentially treated with medicinal plants. Gastrointestinal problems, cough and hypertension were the main illnesses treated by medicinal plants in Beni-Sueif governorate. People may use medicinal plants as an adjuvant therapy to treat these illnesses due to the common occurrence of the former two illnesses and the chronic nature of the latter illness. This may be the reason for why these illnesses have high agreement values. The preservation of the traditional knowledge is an essential requirement for maintaining traditional Egyptian medicine as a cultural resource.

## Competing interests

The authors declare that they have no competing interests.

## Authors' contributions

SA carried out the fieldwork, analysis of the data and drafting the manuscript. AM authenticated the plant samples. All authors read and approved the final manuscript.
